# A Mouse-Tracking Paradigm for Value-Driven Attentional Capture

**DOI:** 10.3390/bs16081273

**Published:** 2026-07-27

**Authors:** Yitong Chen, Fengji Geng, Mengyuan Gong, Hui Zhou, Yuzheng Hu

**Affiliations:** 1 Department of Psychology and Behavioral Sciences, Zhejiang University, Hangzhou 310058, China; 12239031@zju.edu.cn (Y.C.);; 2 Department of Curriculum and Learning Sciences, Zhejiang University, Hangzhou 310058, China; 3 The State Key Lab of Brain-Machine Intelligence, Zhejiang University, Hangzhou 310058, China

**Keywords:** reward learning, value-driven attentional capture, mouse-tracking, reward sensitivity, addiction tendency, individual difference

## Abstract

Reward learning is fundamental to adaptive behavior and exhibits substantial individual variability linked to addiction and other psychiatric conditions. Traditional value-driven attentional capture (VDAC) paradigms rely mainly on reaction time (RT) and accuracy, but it remains unknown whether engaging motor control processes could enhance sensitivity for detecting individual differences in reward-induced attentional biases. We developed a novel mouse-tracking VDAC paradigm that engages motor control processes. Two independent samples (n1 = 45, n2 = 59) completed the task and a battery of reward-related psychological scales (reward sensitivity, impulsivity, self-control, digital addiction tendency). A new index, maximum deviation (MD) of mouse trajectories, quantified how reward history biases attention and motor response. The paradigm replicated the classic RT-based VDAC effect. Trajectory-based measures revealed bidirectional effects: high-value distractors increased attraction and reduced active avoidance. The relative angle modulated VDAC strength, with the largest effect at 120°. ΔMD (high-value vs. absent) showed significant positive correlations with reward sensitivity, impulsivity, and addiction tendency, and negative correlations with self-control in both samples, whereas ΔRT showed no significant correlations. The consistent pattern across two independent samples suggests that mouse-tracking provides a complementary, process-sensitive index for detecting individual differences in reward-driven attention. This paradigm may serve as a useful tool for investigating cognitive markers of vulnerability to reward-related disorders, including addiction.

## 1. Introduction

Reward learning refers to the process by which individuals form associations between specific stimuli and positive outcomes, thereby assigning higher priority to those stimuli in future contexts. This mechanism constitutes a core component of adaptive behavior in both humans and animals, shaping decision-making and motivation while exerting profound influences on early perceptual and attentional processes (Berridge & Robinson, 1998; Hickey et al., 2010; Le Pelley, 2016; Pearson et al., 2022; O’Doherty et al., 2017; Schultz, 1998). A striking demonstration of this phenomenon is the effect of value-driven attentional capture (VDAC), whereby stimuli previously associated with reward continue to attract attention even when they are task-irrelevant (Anderson et al., 2011; Anderson, 2013; Theeuwes & Belopolsky, 2012; Y. Chen et al., 2023). Investigating VDAC is crucial for understanding the exaggerated salience of reward-related cues in addiction (Hyman et al., 2006), as well as the cognitive and behavioral symptoms observed in ADHD and depression (Sali et al., 2018; Whitton et al., 2015). Furthermore, such research is essential for developing interventions that mitigate the disruptive impact of these potent cues (Anderson et al., 2014, 2016; Anderson, 2021; Dayan, 2009).

Despite the extensive use of VDAC paradigms, traditional approaches based on reaction time (RT) and accuracy (ACC) have certain methodological characteristics that may limit their ability to capture the full dynamics of attentional competition. First, these measures primarily reflect the end-point outcome of attentional selection, whereas in naturalistic settings, multiple stimuli compete for limited attentional resources in a continuous and dynamic manner (Draheim et al., 2019; see Stanković, 2025, for a review). Although eye-tracking offers higher temporal resolution (Le Pelley et al., 2015), its application is often constrained by specialized equipment, cost, and scalability. Second, RT-based indices have shown variable sensitivity for assessing individual differences. While group-level effects of VDAC on traits such as addiction severity and impulsivity are well documented, the ability of RT measures to detect fine-grained individual variations has been inconsistent (Albertella et al., 2017, 2019, 2020, 2021; Anderson et al., 2013; Anderson, 2016, 2021; Basel & Lazarov, 2023; Freichel et al., 2023). Additionally, the test-retest reliability of RT indices, although adequate for detecting robust group effects between groups effects, may not always reach the level required for tracking individual differences or within-subject changes over time (Anderson & Kim, 2019; Garre-Frutos et al., 2024; Rusz et al., 2020; Shih et al., 2025). These considerations highlight the value of exploring complementary approaches that can capture continuous, process-level dynamics with greater sensitivity.

Recent advances in understanding the coupling between attention and action offer a promising avenue to overcome these limitations. Cognitive selection has been proposed to unfold as a continuous dynamical process in which multiple competing representations are activated in parallel and partially expressed through the motor system (Spivey & Dale, 2006). Empirical evidence shows that when distractors capture attention, ongoing hand movements (e.g., reaching or mouse trajectories) are transiently deflected toward the distractor before being corrected toward the target (Song & Nakayama, 2009; Freeman & Ambady, 2010). Such trajectory deviations (“partial attraction”) are considered overt traces of attentional competition expressed in the motor system. Accordingly, mouse-tracking methods align well with the dynamic nature of VDAC, providing continuous behavioral indices that can more sensitively capture the unfolding of value-based attentional biases (Egner et al., 2018). Unlike RT and accuracy, which mainly reflect the final outcome of selection, mouse trajectories preserve information about the unfolding competition between the target and distractor during response execution. Recent methodological work (Meyer et al., 2023) suggests that continuous mouse movements can reveal transient cognitive competition that may be missed by endpoint measures alone. Thus, mouse-tracking may provide a process-level complement to conventional VDAC indices rather than simply another measure of response speed. Importantly, computer-based mouse-tracking tasks are relatively low-cost, easy to implement, and suitable for remote data collection.

In the present study, we developed a novel mouse-tracking VDAC paradigm to investigate whether engaging motor control process would yield a more sensitive measure of individual differences in reward-driven attention. Specifically, we have to main aims. First, to validate that mouse trajectories can successfully capture the classic VDAC effect at the group level—that the mouse-trajectory from the start position to the target would bend toward a distractor previously associated with high reward value. Second, to evaluate whether this bending, characterized by maximum deviation (MD) in mouse-trajectory, would reveal individual-difference in reward-related behaviors. We predicted that the degree of bending (i.e., MD) would correlate with the trait of reward sensitivity and reward-related psychologic constructs including impulsivity, self-control, and digital addiction tendency. Finally, we sought to confirm that these findings would replicate consistently across two independent samples, demonstrating the robustness of the mouse-tracking approach.

## 2. Materials and Methods

### 2.1. Participants

The sample size was determined a priori using G*Power 3.1 (Faul et al., 2007). Based on a repeated-measures ANOVA (within factors) with three levels of value condition (High-value, Low-value, Absent), assuming a medium effect size (f = 0.25), α = 0.05, and power = 0.90, the analysis indicated that a minimum of 36 participants was required to detect the main effect of value condition.

*Initial sample.* Forty-six college students were recruited. One participant was excluded due to invalid questionnaire data (failure on lie detection items) and below-chance accuracy (<50%) in the test phase. The final sample comprised 45 participants (27 females and 18 males; 19–28 years of age, *M* = 20.31, *SD* = 2.3).

*Replication sample.* A separate sample of 60 undergraduate and graduate students was recruited. One participant was excluded due to invalid training (accuracy < 50%), resulting in a final sample of 59 participants (41 females and 18 males; 18–28 years of age, *M* = 22.29, *SD* = 2.53).

All participants provided written informed consent in accordance with the ethical guidelines approved by the Institutional Review Board at Zhejiang University. Eligible criteria included normal or corrected-to-normal vision, right-handedness, and no history of neurological or psychiatric disorders. Both samples were recruited from the same university participant pool. Participants were instructed to avoid excessive physical or mental exertion on the day of and the day prior to the experiment, and to ensure adequate sleep to minimize the influence of fatigue or acute stress on behavioral performance. Participants received an average compensation of ¥40, with a portion contingent on their performance in the reward-based training task.

### 2.2. Apparatus

The experiment was programmed in PsychoPy 2024.2.4 with the PyAutoGUI library and presented on a Dell monitor with a resolution of 1920 × 1080 pixels. Participants viewed the display from a distance of approximately 60 cm in a dimly lit room. To control for differences in input devices, all participants used the same model of Dell wired mouse, and the mouse acceleration and system-level smoothing were uniformly disabled prior to the experiment. Mouse positions were recorded in screen coordinates (in pixels) and timestamps (in milliseconds) at a sampling rate of 2000 Hz.

### 2.3. Questionnaires

Prior to the experimental tasks, participants completed an online battery of questionnaires. For the initial sample, addiction tendency was assessed using the Internet Addiction Test (IAT; Young, 1998), the DSM-5 Internet Gaming Disorder checklist (American Psychiatric Association, 2013), and the Social Media Disorder Scale (SMD; Van Den Eijnden et al., 2016). For the replication sample, addiction measures included the Internet Gaming Disorder Test (IGD-20; Pontes et al., 2014; Chinese version by Yu et al., 2019), the DSM-5 Internet Gaming Disorder checklist, the Short Video Indulgence Scale (SVIS; H. Chen et al., 2025), and the DSM-5 Short Video Disorder checklist (adapted version of DSM-5 Internet Gaming Disorder checklist, we substitute the “Internet gaming” with “Short video”). In both samples, Impulsivity was measured with the Barratt Impulsiveness Scale (BIS-11; Patton et al., 1995; Chinese version by Li et al., 2011), and self-control with the Self-Control Scale (SCS; Tangney et al., 2004; Chinese version by Tan & Guo, 2008). Reward sensitivity was assessed using the Sensitivity to Reward subscale (SR) from the Sensitivity to Punishment and Sensitivity to Reward Questionnaire (SPSRQ; Torrubia et al., 2001; Chinese version by Wang, 2012). Detailed information, including number of items, response scales, scoring, and Cronbach’s α are presented in Table 1.

### 2.4. Procedure

Participants completed all questionnaires first, followed by a mouse tracking-based VDAC task adapted from the eye-tracking VDAC paradigm (Anderson & Kim, 2019) and implemented via mouse tracking. The task included 10 practice trials, followed by a formal experiment consisting of a training phase and a test phase, separated by a 10-min rest interval.

**Figure 1:**
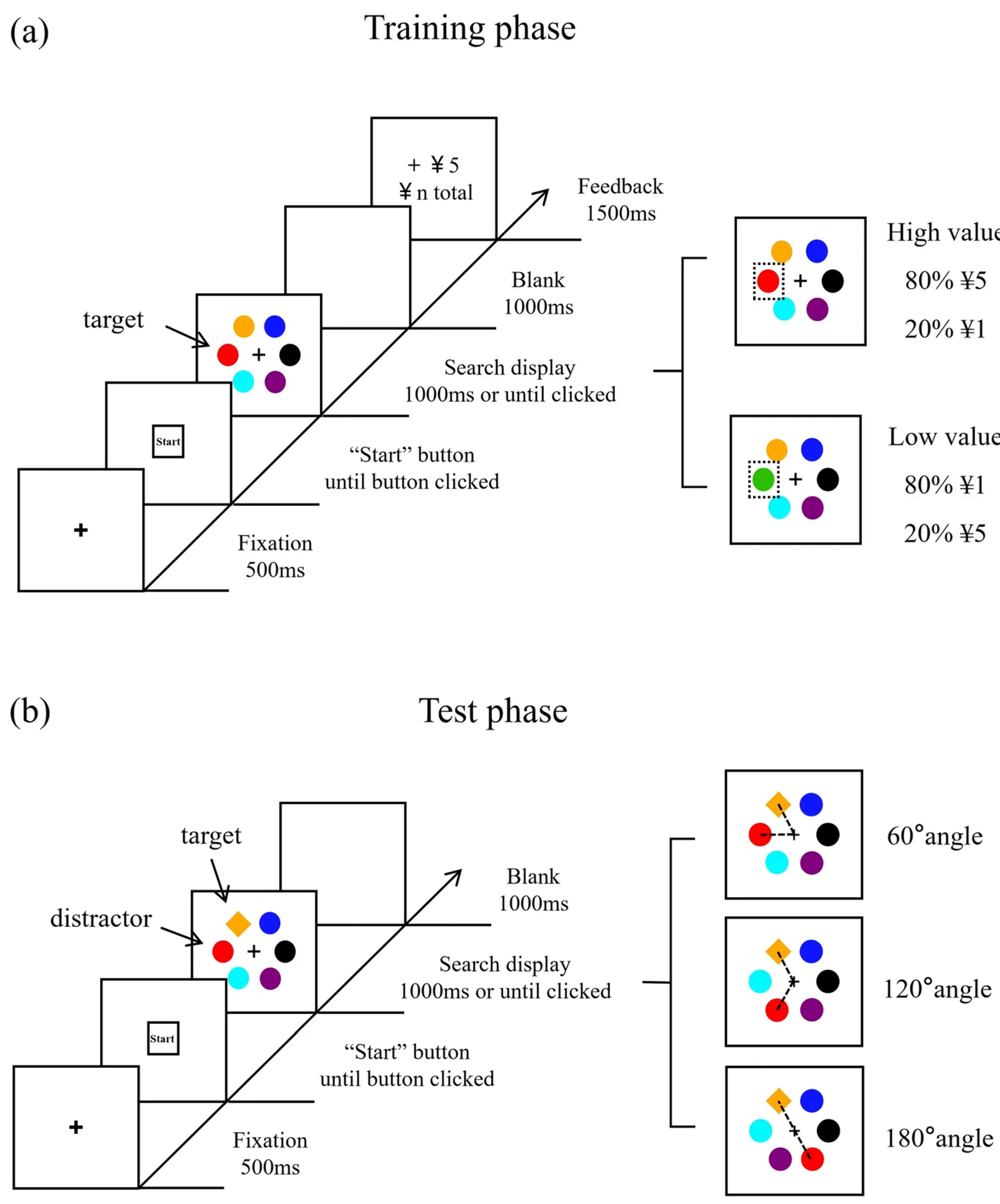
Tasks and Designs for mouse-tracking paradigm. (**a**) In the training phase, participants were instructed to click on the target of a specific color (red or green) with a mouse within 1 s. A correct response was followed by reward feedback, and targets of two different colors corresponded to high/low value. (**b**) In the test phase, participants were instructed to click on the target with a unique shape. The color that formed an association with reward during the training phase appeared as a distractor. No reward feedback was provided. The relative positions of the target and the distractor are categorized into three angle conditions: 60°, 120°, and 180°.

*Training phase.* As shown in Figure 1a, each trial began with the presentation of a central fixation cross for 500 ms, followed by a “Start” button. After clicking the button, the search array was displayed for 1000 ms or until the target was clicked. The search array consisted of six colored circles, each approximately 3.6° of visual angle in diameter, evenly distributed around an imaginary circle with a radius of 10.2° centered on fixation. Participants were instructed to search for a red or green target among six circles. The remaining five distractors were randomly selected from the following colors, with their respective RGB values specified as: blue (0, 0, 255), cyan (0, 255, 255), purple (128, 0, 128), orange (255, 165, 0), yellow (255, 255, 0), and black (0, 0, 0). Participants were required to move the mouse and click on the target within 1000 ms. The search array was followed by a blank screen (1000 ms) and then a feedback screen (1500 ms). The inter-trial interval was 500 ms. If participants correctly identified the target, they received monetary feedback indicating the reward magnitude associated with that color (e.g., “+¥5”) along with their updated cumulative total (e.g., “¥n total”). Missed or incorrect responses resulted in a “Miss” feedback message. Each target color was consistently associated with a fixed reward probability across the training phase: the high-value color had an 80% chance of yielding ¥5 and a 20% chance of yielding ¥1, while the low-value color followed the opposite schedule. For half of the participants, red served as the high-value color; for the other half, green served as the high-value color. Importantly, participants were not informed of these color–reward contingencies but were encouraged to perform as well as possible to maximize their total payoff. The final payment was determined based on their final accumulated reward in this phase, with a maximum of ¥45. The training phase consisted of 4 blocks, each containing 60 trials.

*Test phase.* As shown in Figure 1b, the task structure remained similar to the test phase except that the target was now defined by a unique shape (i.e., a diamond among circles or a circle among diamonds, with both trial types occurring equally often). Correct responses no longer yielded monetary feedback, although incorrect responses still produced the “Miss” feedback. In this phase, stimulus colors were irrelevant to the task, and participants were instructed to ignore color information. However, the colors previously associated with reward during training reappeared as task-irrelevant distractor. On one-third of the trials, the distractor appeared in the previously high-value color (high-value distractor trials); on another one-third, it appeared in the previously low-value color (low-value distractor trials); and in the remaining one-third, no previously reward-associated color was presented (absent-distractor trials). The positions of targets and distractors were fully counterbalanced across the six possible locations. The test phase consisted of 3 blocks, each containing 90 trials.

### 2.5. Data Analysis

First, we calculated the mean reaction time (RT) and accuracy (ACC) under each experimental distractor condition (high-value, low-value, and absent) for both the training phase and the test phase in order to characterize how value shapes learning. Only trials with correct responses were included for RT calculations, while trials involving incorrect responses or time-out responses were excluded.

**Figure 2:**
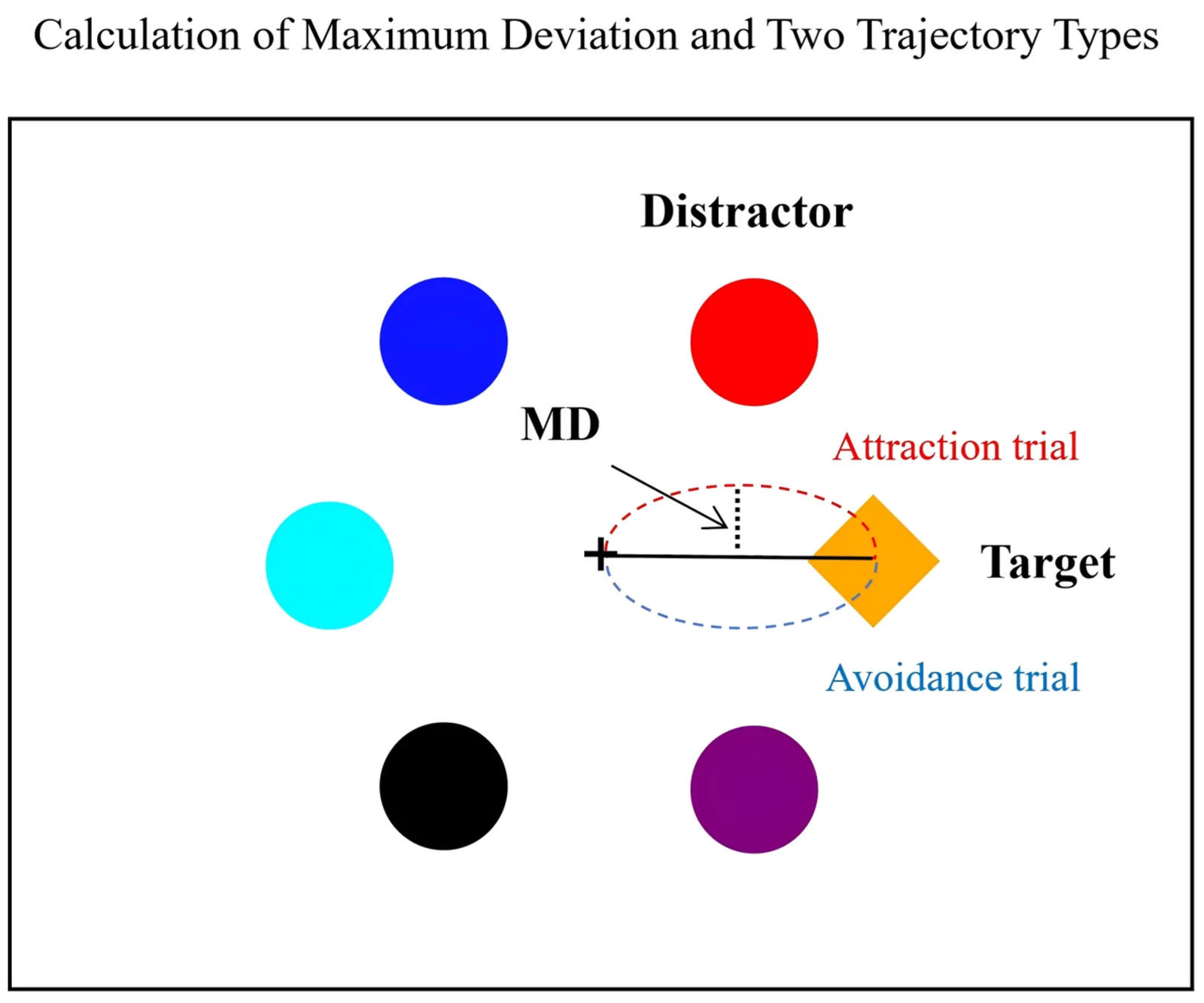
Schematic illustration of Maximum deviation (MD) calculation. The black solid line represents the ideal straight path from the start point to the selected target. The red and blue dashed lines represent two possible patterns of observed mouse trajectories: red lines denote attraction trajectories that curve toward the distractor, whereas blue lines indicate avoidance trajectories deviating from the distractor. Maximum Deviation (MD), indicated by the black dashed perpendicular line, is the greatest distance between the observed trajectory and the ideal path, quantifying peak attraction to the distractor.

Next, we computed mouse trajectory indices for all correct trials in test phase using the Maximum Deviation (MD) metric (Figure 2). MD is the maximum perpendicular distance between the observed trajectory and the idealized straight path from the start to the target positions, indexing peak, momentary attraction toward an unchosen alternative and thus reflecting maximum instantaneous competition during the trial. This metric is widely used to capture response competition or partial co-activation of competing representations (Spivey et al., 2005; Freeman & Ambady, 2010; Hehman et al., 2015).

To ensure comparability across trials, we applied common preprocessing steps following established practices (e.g., Freeman & Ambady, 2010; Kieslich & Henninger, 2017). Specifically, all trajectories were time-normalized to 101 time steps and spatially remapped to a standardized coordinate space. Normalized trajectories were then grouped by distractor value (high-value, low-value, absent) and target-distractor angle (60°, 120°, 180°; see Figure 1b) for subsequent analyses. The three angular separations were selected to sample distinct target-distractor configurations while keeping the display geometry tractable and comparable across conditions. Specifically, 60° represented a relatively adjacent configuration, 120° represented an intermediate non-adjacent configuration, and 180° represented the opposite-location configuration in which the target and distractor were maximally separated. By spanning adjacent, intermediate, and opposite-location configurations, this design allowed us to examine whether trajectory deviations varied with target-distractor geometry, including possible differences related to visual-field arrangement and motor constraints during movement execution.

To further characterize the direction of trajectory bias, trials were classified as attraction or avoidance trials based on the position of the peak deviation relative to the distractor. For each trial, we first defined the ideal straight path from the start position to the target, which served as the reference line. We then determined whether the point of maximum deviation and the distractor were located on the same side of this line. Trials were classified as attraction trials when the peak deviation and distractor were on the same side of the reference line, and as avoidance trials when they were on opposite sides. Thus, attraction and avoidance were defined by the direction of the peak deviation relative to the distractor, whereas the magnitude of this deviation was quantified by MD. Trials with MD values beyond ±3 standard deviations from the participant’s mean were excluded as outliers.

To obtain a composite measure of addiction tendency, we conducted principal component analysis (PCA) separately for the initial and replication samples on the addiction questionnaires administered in each sample (initial: IAT, DSM-5 game, SMD; replication: SVIS, DSM-5 video, IGD-20, DSM-5 game). In line with our aim of capturing a general addiction proneness factor, we extracted the first principal component for each sample for subsequent correlational analyses.

To assess specificity and sensitivity of VADC effects in characterizing individual differences in reward related traits, we examined the correlation between participants’ questionnaire scores and VDAC indices, ΔRT and ΔMD. The ΔRT refers to the changes of participants’ reaction time of trials during the test phase when distractor previously associated with high-value presented (high-value trials), compared to that without a distractor (absent trials). The ΔMD refers to the change in participants’ mean MD on high-value trials compared to absent trials in test phase. Pearson correlation coefficients were calculated, and *p*-values were adjusted for multiple comparisons using the Bonferroni method.

## 3. Results

### 3.1. Training Phase: Reward Learning Effectiveness

To validate the effectiveness of the reward-learning manipulation during the training phase, we conducted paired-sample *t*-tests on reaction time (RT) and accuracy (ACC).

**Figure 3:**
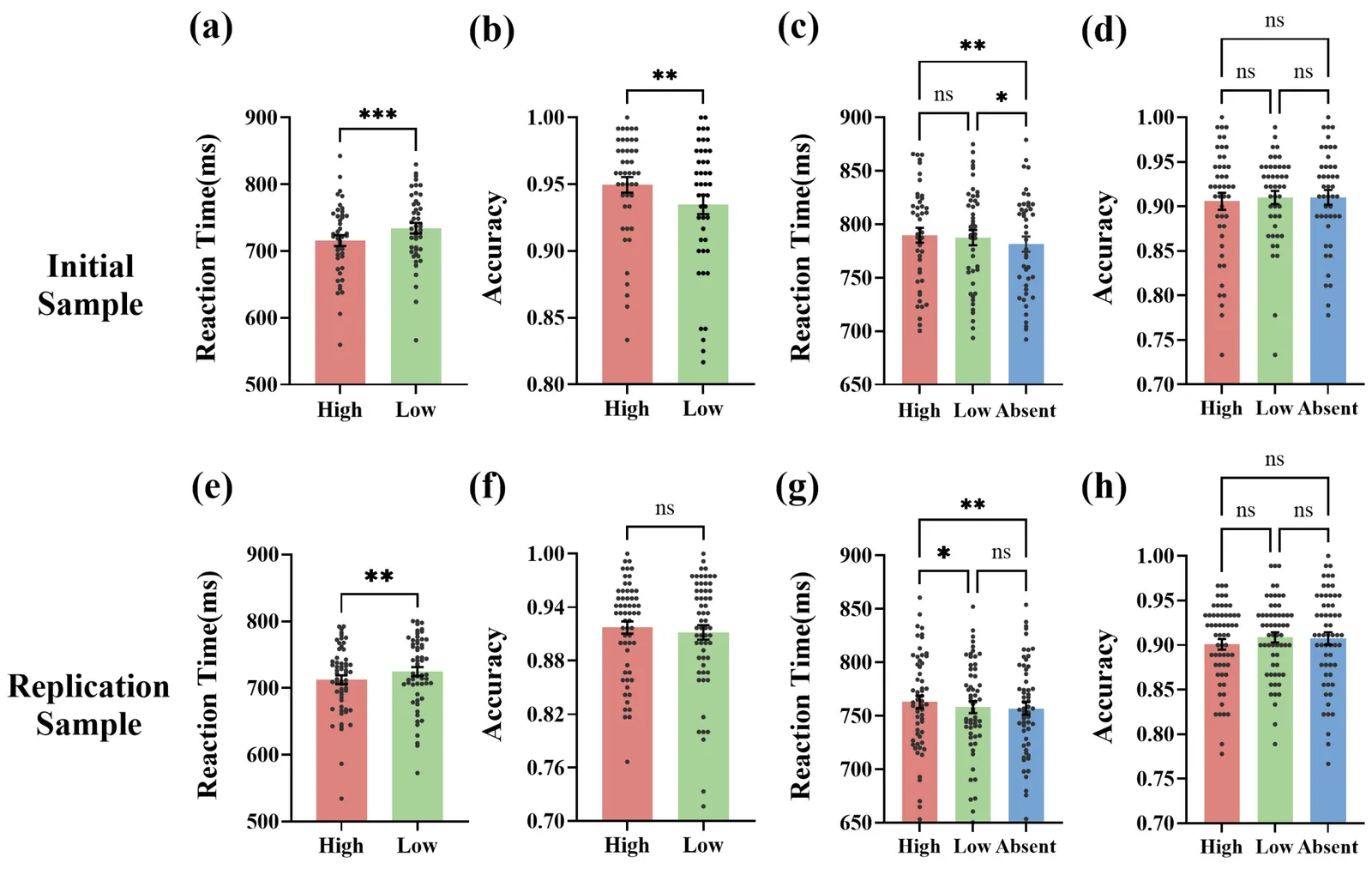
RT and ACC results in the training and test phases. The upper panels present results from the initial sample, and the lower panels present results from the replication sample. Panels (**a**,**e**) and (**b**,**f**) show RT and ACC data from the training phase, while Panels (**c**,**g**) and (**d**,**h**) show RT and ACC data from the test phase. Error bars reflect the within-subjects S.E.M. * *p* < 0.05, ** *p* < 0.01, *** *p* < 0.001, ns: not significant.

*Initial sample.* Participants responded significantly faster to high-value targets than to low-value targets (high: *M* = 716 ms, *SD* = 55 ms; low: *M* = 734 ms, *SD* = 54 ms; *t*_(44)_ = 4.33, *p* < 0.001, Cohen’s *d* = 0.65, Figure 3a). Accuracy was also significantly higher for high-value targets than for low-value targets (high: *M* = 0.95, *SD* = 0.04; low: *M* = 0.94, *SD* = 0.05; *t*_(44)_ = 2.84, *p* = 0.007, Cohen’s *d* = 0.42, Figure 3b).

*Replication sample.* RT was significantly shorter for high-value than for low-value targets (high: *M* = 712 ms, *SD* = 51 ms; low: *M* = 725 ms, *SD* = 52 ms; *t*_(58)_ = 2.81, *p* = 0.007, Cohen’s *d* = 0.37, Figure 3e). Accuracy was numerically higher for high-value than for low-value targets (high: *M* = 0.92, *SD* = 0.05; low: *M* = 0.91, *SD* = 0.06), but this difference did not reach significance (*t*_(58)_ = 0.75, *p* = 0.46, Cohen’s *d* = 0.10) (Figure 3f).

These results confirm the effectiveness of the reward learning manipulation in both samples: participants successfully learned the color-reward associations, exhibiting more efficient behavioral responses to high-value stimuli.

### 3.2. Test Phase: VDAC Effects on Reaction Time and Accuracy

To examine VDAC in the test phase, we conducted separate one-way repeated-measures ANOVAs on RT and ACC, with distractor condition (high-value/low-value/absent) as the within-subject factor.

*Initial sample.* For RT, the ANOVA revealed a significant main effect of condition (*F*_(2, 88)_ = 8.61, *p* < 0.001, *η*_p_^2^ = 0.16, Figure 3c). Post-hoc comparisons showed that RT was significantly slower in the high-value condition than in the absent condition (high: *M* = 789.7 ms, *SD* = 46.5 ms; absent: *M* = 781.3 ms, *SD* = 47.3 ms, *t*_(44)_ = 3.77, *p* = 0.002, Cohen’s *d* = 0.18). RT was also slower in the low-value condition than in the absent condition (low: *M* = 787.5 ms, *SD* = 47.9 ms, *t*_(44)_ = 2.89, *p* = 0.018, Cohen’s *d* = 0.13). No significant difference was found between high-value and low-value conditions (*t*_(44)_ = 1.14, *p* = 0.778, Cohen’s *d* = 0.05). For ACC, the main effect of condition was not significant (*F*_(2, 88)_ = 0.32, *p* = 0.729, *η*_p_^2^ = 0.01), and none of the pairwise comparisons reached significance (all *p* > 0.05) (Figure 3d).

*Replication sample*. The main effect of condition on RT was significant (*F*_(2, 116)_ = 6.36, *p* = 0.002, *η*_p_^2^ = 0.10, Figure 3g). Post-hoc comparisons indicated that RT was significantly slower in the high-value condition than in the low-value condition (high: *M* = 762.9 ms, *SD* = 45.23 ms; low: *M* = 758.1 ms, *SD* = 43.5 ms; *t*_(58)_ = 2.50, *p* = 0.046, Cohen’s *d* = 0.11), and than in the absent condition (*M* = 756.9 ms, *SD* = 45.68 ms; *t*_(58)_ = 3.75, *p* = 0.001, Cohen’s *d* = 0.13). No difference was observed between low-value and absent condition (*t*_(58)_ = 0.67, *p* = 1.000, Cohen’s *d* = 0.03). For ACC, the main effect of condition was not significant (*F*_(2, 116)_ = 1.20, *p* = 0.306, *η*_p_^2^ = 0.02; all paired *t*-test *p* > 0.05) (Figure 3h).

Together, these findings demonstrate that both high-value and low-value distractors capture attention, with this capture primarily reflected in slowed RT rather than reduced ACC.

### 3.3. Trajectory-Based Measures of Attraction and Avoidance

To examine the overall pattern of attraction and avoidance trajectories in the test phase, we analyzed the proportion of attraction trials across distractor conditions and the MD values for both trajectory types using separate one-way repeated-measures ANOVAs.

**Figure 4:**
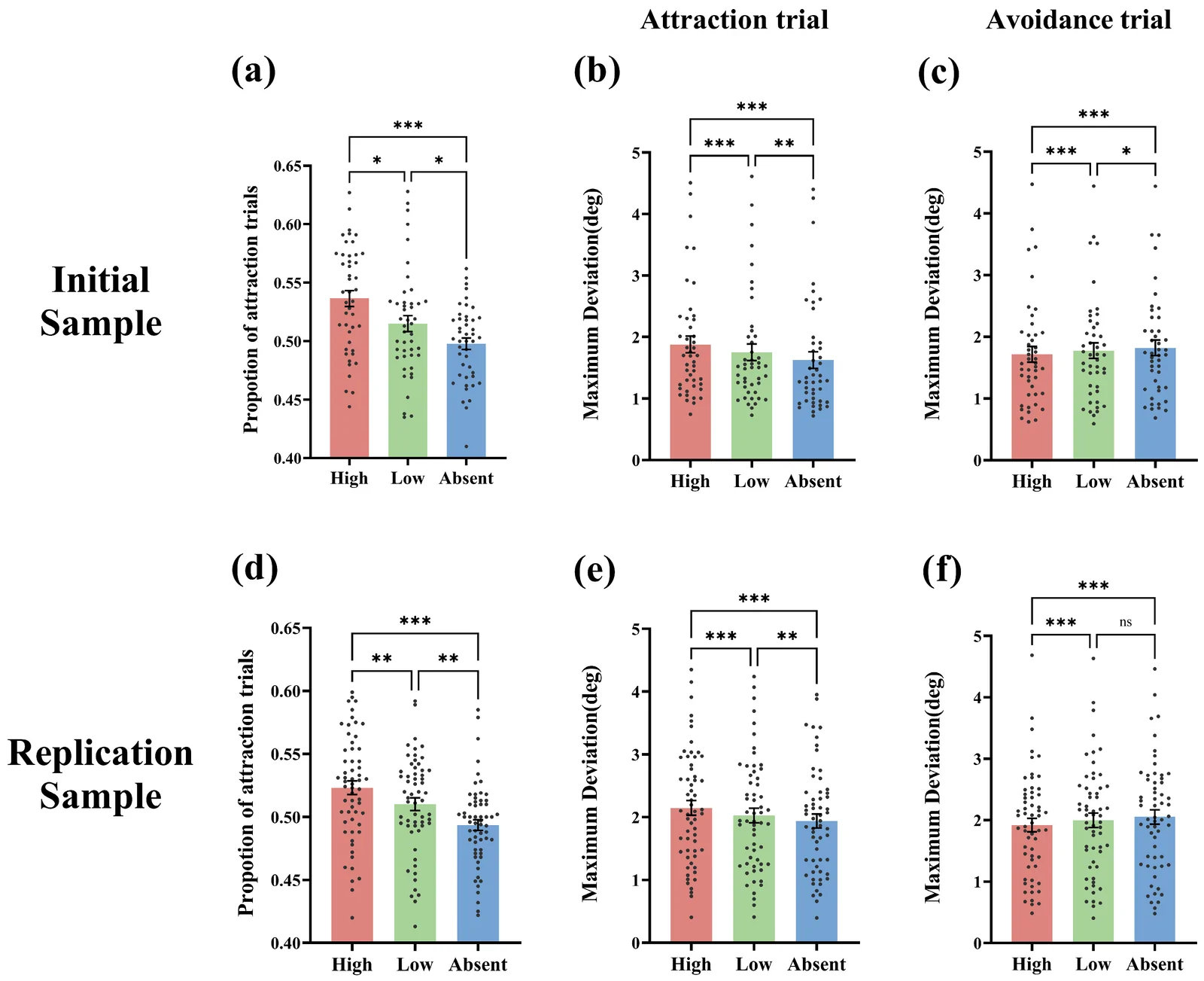
Results of two types of trajectory analyses during the test phase. The upper panels show results from the initial sample, and the lower panels show results from the replication sample. Panels (**a**,**d**) presents the proportion of attraction trials. Panels (**b**,**e**) shows the mean MD values for attraction trials across the three distractor conditions. Panels (**c**,**f**) shows the mean MD values for avoidance trials across the three conditions. Error bars reflect the within-subjects S.E.M. * *p* < 0.05, ** *p* < 0.01, *** *p* < 0.001, ns: not significant.

*Initial sample.* The proportion of attraction trials differed significantly across conditions (*F*_(2, 88)_ = 15.13, *p* < 0.001, *η*_p_^2^ = 0.26), with a higher proportion in the high-value condition than in the low-value and absent conditions (high: *M* = 0.54, *SD* = 0.05; low: *M* = 0.52, *SD* = 0.05; absent: *M* = 0.50, *SD* = 0.03; high vs. low: *t*_(44)_ = 2.84, *p* = 0.021, Cohen’s *d* = 0.51; high vs. absent: *t*_(44)_ = 5.69, *p* < 0.001, Cohen’s *d* = 0.93; Figure 4a). For attraction trials, the ANOVA on MD revealed a significant main effect of condition (*F*_(2, 88)_ = 30.29, *p* < 0.001, *η*_p_^2^ = 0.41). MD was significantly larger for high-value than for low-value (high: *M* = 1.88, *SD* = 0.91; low: *M* = 1.75, *SD* = 0.90; *t*_(44)_ = 5.57, *p* < 0.001, Cohen’s *d* = 0.15) and absent (*M* = 1.65, *SD* = 0.90; *t*_(44)_ = 7.05, *p* < 0.001, Cohen’s *d* = 0.27; Figure 4b). For avoidance trials, the main effect of condition was also significant (*F*_(2, 88)_ = 17.56, *p* < 0.001, *η*_p_^2^ = 0.29), but the pattern was reversed: absent trials elicited larger MD than low-value and high-value trials (absent: *M* = 1.82, *SD* = 0.84; low: *M* = 1.78, *SD* = 0.84; high: *M* = 1.72, *SD* = 0.85; absent vs. low: *t*_(44)_ = 2.56, *p* = 0.042, Cohen’s *d* = 0.05; absent vs. high: *t*_(44)_ = 5.13, *p* < 0.001, Cohen’s *d* = 0.12; Figure 4c).

*Replication sample.* The pattern replicated. The main effect of condition on the proportion of attraction trials was significant (*F*_(2, 116)_ = 24.39, *p* < 0.001, *η*_p_^2^ = 0.30), with a higher proportion for high-value than for low-value (high: *M* = 0.52, *SD* = 0.04; low: *M* = 0.51, *SD* = 0.04; *t*_(58)_ = 3.73, *p* = 0.001, Cohen’s *d* = 0.34), and absent (*M* = 0.49, *SD* = 0.03; *t*_(58)_ = 6.51, *p* < 0.001, Cohen’s *d* = 0.78; Figure 4d). For attraction trials, the main effect of condition on MD was significant (*F*_(2, 116)_ = 31.52, *p* < 0.001, *η*_p_^2^ = 0.35). MD was larger for high-value than for low-value (high: *M* = 2.14, *SD* = 0.89; low: *M* = 2.05, *SD* = 0.90; *t*_(58)_ = 4.66, *p* < 0.001, Cohen’s *d* = 0.10) and absent (*M* = 1.96, *SD* = 0.86; *t*_(58)_ = 7.16, *p* < 0.001, Cohen’s *d* = 0.20; Figure 4e). For avoidance trials, the main effect of condition was also significant (*F*_(2, 116)_ = 18.22, *p* < 0.001, *η*_p_^2^ = 0.24), with absent trials showing larger MD than low-value (absent: *M* = 2.06, *SD* = 0.90; low: *M* = 2.00, *SD* = 0.88; marginally, *t*_(58)_ = 2.34, *p* = 0.068, Cohen’s *d* = 0.06) and high-value (*M* = 1.92, *SD* = 0.84; *t*_(58)_ = 5.23, *p* < 0.001, Cohen’s *d* = 0.15; Figure 4f).

Together, both samples indicate that high-value and low-value distractors not only elicit positive attraction but also reduce avoidance, providing complementary evidence for the VDAC effect.

### 3.4. Modulation by Target-Distractor Angle

To examine the role of target-distractor angle, we conducted a two-way repeated-measures ANOVA on MD from attraction trials, with angle (60°, 120°, 180°) and distractor condition (high-value, low-value, absent) as within-subject factors.

**Figure 5:**
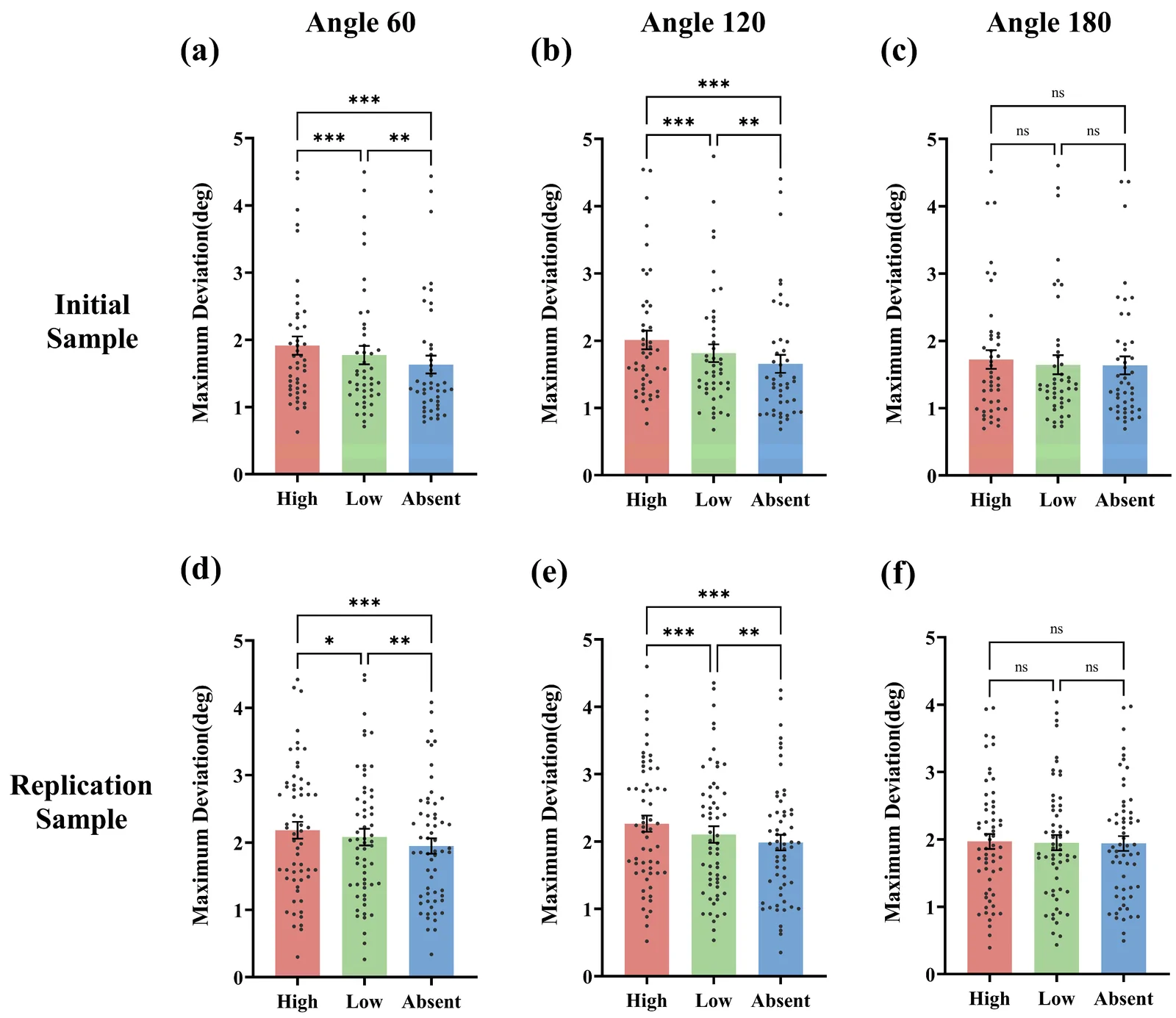
Results of the interaction between Condition and Angle for attraction trials in the test phase. The upper panels show results from the initial sample, and the lower panels show results from the replication sample. Panels (**a**,**d**), (**b**,**e**), and (**c**,**f**) present the mean MD values at angle levels of 60°, 120°, and 180°, respectively, across the three experimental conditions. Error bars reflect the within-subjects S.E.M. * *p* < 0.05, ** *p* < 0.01, *** *p* < 0.001, ns: not significant.

*Initial sample.* Sphericity was violated for the angle factor (*p* = 0.02), so Greenhouse-Geisser correction was applied. The main effect of angle (*F*_(1.714, 75.42)_ = 20.66, *p* < 0.001, *η*_p_^2^ = 0.32) and the angle × condition interaction (*F*_(4, 176)_ = 7.54, *p* < 0.001, *η*_p_^2^ = 0.15) were significant. Post-hoc comparisons revealed that MD at 120° was significantly larger than at 60° (*t*_(44)_ = 2.77, *p* = 0.025, Cohen’s *d* = 0.06) and at 180° (*t*_(44)_ = 3.77, *p* = 0.001, Cohen’s *d* = 0.12). The main effect of condition was significant at 60° (*F*_(2, 88)_ = 22.13, *p* < 0.001, *η*_p_^2^ = 0.34; Figure 5a) and 120° (*F*_(2, 88)_ = 44.34, *p* < 0.001, *η*_p_^2^ = 0.50; Figure 5b), but not at 180° (*F*_(2, 88)_ = 2.01, *p* = 0.141, *η*_p_^2^ = 0.04, Figure 5c). At 60°, high-value MD was larger than low-value and absent (high: *M* = 1.91, *SD* = 0.91; low: *M* = 1.77, *SD* = 0.92; absent: *M* = 1.63, *SD* = 0.89; high vs. low: *t*_(44)_ = 3.94, *p* < 0.001, Cohen’s *d* = 0.15; high vs. absent: *t*_(44)_ = 6.02, *p* < 0.001, Cohen’s *d* = 0.31). The same pattern held at 120° (high: *M* = 2.01, *SD* = 0.93; low: *M* = 1.82, *SD* = 0.89; absent: *M* = 1.66, *SD* = 0.90; high vs. low: *t*_(44)_ = 6.06, *p* < 0.001, Cohen’s *d* = 0.22; high vs. absent: *t*_(44)_ = 9.38, *p* < 0.001, Cohen’s *d* = 0.39), with larger effect sizes than at 60°.

*Replication sample*. Sphericity was violated for the angle factor (*p* < 0.001) and the interaction (*p* = 0.027), so Greenhouse-Geisser correction was applied. The main effect of angle (*F*_(1.446, 83.88)_ = 14.36, *p* < 0.001, *η*_p_^2^ = 0.20) and the condition × angle interaction (*F*_(3.406, 197.6)_ = 11.03, *p* < 0.001, *η*_p_^2^ = 0.16) were significant. Post-hoc comparisons showed that MD at 120° was larger than at 60° (marginally, *t*_(58)_ = 2.35, *p* = 0.067, Cohen’s *d* = 0.05) and significantly larger than at 180° (*t*_(58)_ = 3.19, *p* = 0.007, Cohen’s *d* = 0.13). The main effect of condition was significant at 60° (*F*_(2, 116)_ = 22.03, *p* < 0.001, *η*_p_^2^ = 0.28; Figure 5d) and 120° (*F*_(2, 116)_ = 31.40, *p* < 0.001, *η*_p_^2^ = 0.35; Figure 5e), but not at 180° (*F*_(2, 116)_ = 0.70, *p* = 0.500, *η*_p_^2^ = 0.01; Figure 5f). Again, the largest effect sizes were observed at 120° (high vs. low: *t*_(58)_ = 4.86, *p* < 0.001, Cohen’s *d* = 0.17; high vs. absent: *t*_(58)_ = 7.06, *p* < 0.001, Cohen’s *d* = 0.30).

These results indicate that, relative to the absent condition, distractors increased MD, reflecting attentional capture. High-value distractors elicited larger MD than low-value distractors, confirming VDAC at the trajectory level. Moreover, the target-distractor angle significantly modulated VDAC: the effect was robust at 60° and 120° but absent at 180°, with the strongest capture observed at 120°.

### 3.5. Individual Differences: Correlations with Psychological Traits

For the initial sample, the Kaiser-Meyer-Olkin (KMO) measures were 0.577 (IAT), 0.652 (DSM-5 game), and 0.641 (SMD), and Bartlett’s test of sphericity was significant (*χ*^2^(3) = 34.29, *p* < 0.001). The extracted first principal component had an eigenvalue of 1.959 and explained 65.3% of the total variance, with component loadings of 0.883 (IAT), 0.774 (DSM-5 game), and 0.762 (SMD). For the replication sample, the KMO values were 0.615 (SVIS), 0.585 (DSM-5 video), 0.546 (IGD-20), and 0.571 (DSM-5 game), and Bartlett’s test was significant (*χ*^2^(6) = 118.98, *p* < 0.001). The extracted first principal component had an eigenvalue of 2.260 and explained 56.5% of the variance, with loadings of 0.830 (SVIS), 0.764 (DSM-5 video), 0.714 (IGD-20), and 0.692 (DSM-5 game). These consistently high loadings suggest that the component can be interpreted as a general addiction tendency factor.

**Figure 6:**
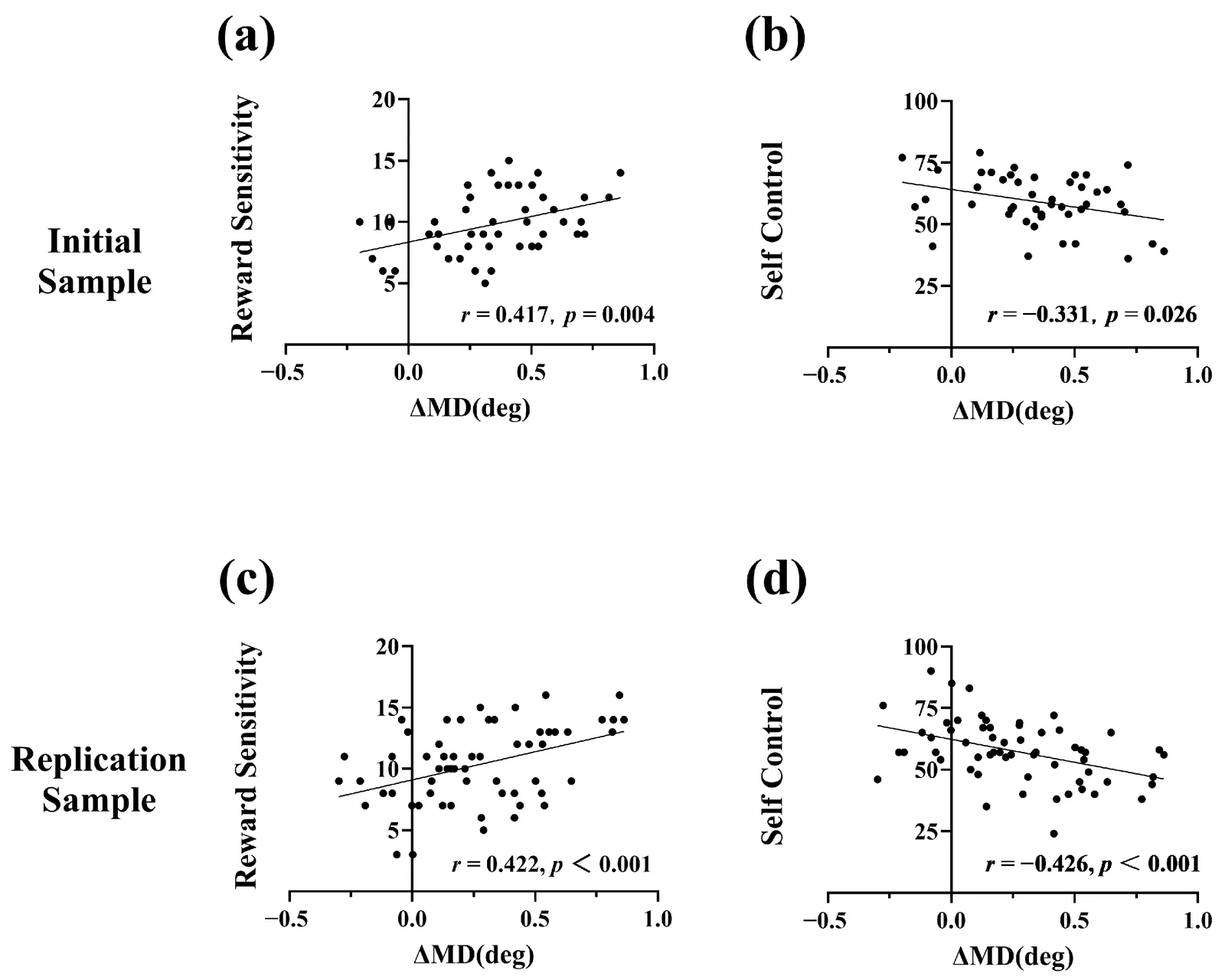
Scatter plots for correlation analysis. The upper panels present results from the initial sample, and the lower panels from the replication sample. Panels (**a**,**c**) show the correlation between the mouse-tracking VDAC effect (ΔMD) and reward sensitivity scores (Reward subscale of the Sensitivity to Reward Questionnaire, SR). Panels (**b**,**d**) show the correlation between the same index and self-control scores (Self-Control Scale, SCS).

To examine associations with VDAC, we calculated Pearson correlations between the VDAC indices (ΔRT and ΔMD) and questionnaire scores. In the initial sample, ΔMD was significantly positively correlated with SR (Figure 6a), IAT, DSM-5 game, PCA addiction tendency, and BIS-11, and negatively correlated with SCS (Figure 6b), whereas ΔRT showed no significant correlations. Similar patterns were observed in the replication sample, with ΔMD significantly correlated with SR (Figure 6c), DSM-5 game, PCA addiction tendency, BIS-11, and SCS (Figure 6d). Detailed correlational findings are presented in Table 2 and Table 3.

## 4. Discussion

In this study, we developed and validated a mouse-tracking paradigm for value-driven attentional capture (VDAC) across two independent samples. The results showed that mouse-tracking indices replicated the classic VDAC effect and revealed individual differences that were not captured by traditional reaction time (RT) measures. Specifically, reward sensitivity, impulsivity, and addiction-related traits were positively correlated with the trajectory-based VDAC effect (ΔMD), whereas self-control was negatively correlated with ΔMD. These findings support the view that mouse tracking provides a process-sensitive behavioral index of reward-driven attentional competition.

### 4.1. Bidirectional Trajectory Effects of Reward History

Movement trajectory analyses revealed a bidirectional effect of reward history. High-value distractors elicited a higher proportion of attraction trials and larger maximum deviation (MD) than absent-distractor trials. At the same time, they reduced active avoidance, as indicated by smaller MD on avoidance trials. This pattern suggests that previously rewarded distractors not only increased the pull of attention and action toward the distractor, but also weakened the tendency to move away from it. Thus, reward learning appears to shape both attraction and avoidance components of attentional competition.

These findings are consistent with incentive-sensitization theory (Robinson & Berridge, 1993), which proposes that reward-associated stimuli acquire incentive salience and become more likely to attract attention. They also support the view of attention as a competitive and dynamically unfolding process (Spivey & Dale, 2006). RTs reflect the final outcome of this competition, whereas mouse trajectories preserve information about the intermediate pull of competing stimuli during response execution.

This interpretation also connects the present paradigm with oculomotor measures of attentional competition. Previous work has shown that saccade trajectories can deviate toward or away from distractors, providing a sensitive index of distractor processing during attentional selection (Dalmaso, 2022). Mouse trajectories may reflect a related form of competition, but expressed through manual action rather than eye movements. The two methods therefore provide complementary information: eye movements offer a more direct and temporally precise measure of overt visual orienting, whereas mouse tracking captures how attentional competition is integrated into action planning and response execution. This makes mouse tracking a low-cost and scalable complement to eye-tracking and saccade-based measures, especially in large-sample or remote testing contexts.

### 4.2. Spatial Modulation by Target-Distractor Angle

The VDAC effect was significant at both 60° and 120° but was consistently stronger at 120°, as indicated by larger effect sizes. No reliable capture was observed at 180°. This pattern suggests that reward-driven attentional competition is spatially tuned. A distractor at 120° may provide an especially sensitive condition because it is far enough from the target to avoid simple overlap with the target-directed response but close enough to bias the unfolding movement trajectory. At 60°, the distractor was adjacent to the target and may have been more strongly constrained by local target selection or spatial suppression. At 180°, the distractor was opposite the target and may have exerted less influence on the target-directed movement.

The use of 60°, 120°, and 180° also allowed us to sample three distinct spatial relations within the six-location circular array while keeping target and distractor positions fully counterbalanced. It is worth noting that all stimuli were positioned well within the display boundaries (radius of approximately 10.2° of visual angle), with no stimuli appearing near the monitor edges. Thus, the absence of VDAC at 180° is unlikely to reflect physical compression of motor trajectories by visual field boundaries, but rather the spatial dynamics of attentional competition when the target and distractor are maximally separated. Therefore, the present angle effect should be interpreted as evidence that the spatial relation between target and distractor modulates trajectory-based VDAC, rather than as evidence that 120° is a universally optimal angle across all task layouts. Future studies could test finer angular separations and examine whether the same spatial tuning pattern generalizes to other display geometries and response demands.

### 4.3. Trajectory-Based VDAC and Individual Differences

Although RT also showed a significant VDAC effect, its effect size was relatively modest. More importantly, the mouse-tracking index ΔMD (the increase in MD on high-value vs. absent trials) was significantly positively correlated with reward sensitivity (SR), impulsivity (BIS-11), and addiction tendency, and significantly negatively correlated with self-control (SCS) in both samples. In contrast, ΔRT showed no significant correlations with any of these trait measures. This dissociation suggests that ΔMD captures individual difference-relevant variance that ΔRT dose not. The consistent pattern observed across two independent samples aligns with recent calls for using motor-dynamics measures in cognitive assessment (Smith et al., 2022).

The present findings suggest that reward-related attentional bias is not only expressed as delayed responses but also as a measurable pull in the ongoing motor trajectory. This may be especially useful when individual differences are subtle and may not produce large RT effects.

In future work, this mouse-tracking paradigm could complement existing RT-based VDAC tasks by providing a continuous index of how reward-related distractors influence response dynamics. Its low equipment requirements may make it suitable for large-sample studies, longitudinal designs, and online assessment. However, ΔMD should not be treated as a diagnostic marker on its own. Rather, it may help characterize individual variation in reward-related attentional bias when used together with self-report, clinical assessment, and other behavioral or physiological measures.

### 4.4. Theoretical Links to Psychological Traits

The associations between VDAC strength and psychological traits are consistent with the idea that individuals who are more sensitive to reward show stronger attentional bias toward previously rewarded cues. The positive correlation with reward sensitivity suggests that people who are more responsive to rewarding outcomes may assign greater attentional priority to reward-associated stimuli (Kim et al., 2015). This finding aligns with theories emphasizing individual differences in stimulus-reward learning and incentive salience attribution (Flagel & Robinson, 2017).

The positive correlations with impulsivity and addiction-related traits further suggest that stronger reward-driven attentional capture may be linked to difficulty resisting reward-related cues. This is broadly consistent with prior work showing that reward-related attentional capture is associated with addictive and compulsive behaviors (Albertella et al., 2019). Conversely, the negative correlation between self-control and ΔMD highlights the possible protective role of cognitive control. Individuals with higher self-control may be better able to limit the influence of irrelevant reward-associated distractors, thereby showing weaker trajectory-based capture.

It is important to define the scope of this interpretation. The addiction-related measures used in the present study mainly assessed digital behaviors, including internet use, gaming, social media, and short-video use. Therefore, the PCA-derived component should be interpreted as a general digital addiction tendency rather than as a broad addiction factor covering substance-use disorders. Although incentive-sensitization theory was originally developed in the context of substance addiction, reward cue-reactivity and attentional bias have also been discussed in behavioral addictions (e.g., Dong & Potenza, 2014; Starcke et al., 2018). The present findings should therefore be viewed as extending this framework to digital behavior-related traits.

### 4.5. Possible Neural Mechanisms and Future Directions

The observed association between VDAC and individual differences may be related to dopamine-induced plasticity (Holroyd & Coles, 2002; Keiflin & Janak, 2015; Mollick et al., 2021; Schultz, 2016). Repeated pairing of a stimulus with reward triggers dopamine release in the nucleus accumbens and related circuits, increasing the incentive salience of that stimulus (Berridge & Robinson, 1998). Consistent with this possibility, prior work has implicated dopamine in value-based attentional orienting (Anderson et al., 2016), suggesting that dopaminergic mechanisms may contribute to the acquisition or expression of attentional priority for reward-associated stimuli. Individual variations in this dopaminergic response (arising from genetic or experiential factors) may partly explain why some individuals show stronger reward-driven attentional capture.

Mouse-tracking deviations offer a continuous, real-time readout of such motivational processes. Together with traditional RT measures, which reliably capture the outcome of attentional competition, trajectory-based indices may provide complementary insight into the phasic dynamics of incentive salience. However, the present study did not include neural, pharmacological, or physiological measures. Therefore, the current findings should be interpreted as behavioral evidence for individual differences in reward-driven attentional competition, and the dopaminergic account remains a possible mechanism to be tested in future work. Future studies could combine mouse tracking with EEG, fMRI, pharmacological manipulations, or computational modeling to examine how reward-learning signals are translated into attentional and motor competition, and may also consider more distal biological mechanisms, such as altered RNA processing or alternative splicing (Ran et al., 2026), that could contribute to individual differences in neural plasticity and reward-circuit function.

### 4.6. Summary and Limitations

In summary, this study applied mouse tracking to VDAC research and showed that trajectory-based measures can capture reward-driven attentional competition in a continuous manner. Across two independent samples, high-value distractors increased attraction and reduced avoidance in mouse trajectories. The effect was modulated by target-distractor angle and was strongest at 120°. Most importantly, ΔMD was associated with reward sensitivity, impulsivity, self-control, and digital addiction tendency, whereas ΔRT was not. These findings suggest that engaging motor control can reveal individual differences in reward-driven attention that may be less evident in traditional endpoint measures.

Several limitations should be acknowledged. First, both samples consisted of healthy university students from the same university, which limits generalizability to broader populations. High-functioning young adults may differ from individuals with diagnosed addictions in baseline reward processing, dopaminergic functioning, and cognitive control. Second, the study was cross-sectional and correlational, so it cannot determine whether stronger trajectory-based VDAC is a cause, consequence, or correlate of addiction-related traits. Third, although the equipment was standardized, individual differences in mouse handling may still contribute to trajectory variability. Fourth, the addiction-related measures focused mainly on digital behaviors rather than substance use. Future studies should examine whether the present trajectory-based index generalizes to substance-related disorders and clinically diagnosed behavioral addictions. Fifth, the replication sample showed a significant RT advantage for high-value targets during training, but the corresponding accuracy difference was only numerical. This pattern does not necessarily indicate a failure of reward learning, because reward-learning effects in VDAC tasks are often more reliably expressed in response speed than in accuracy, particularly when overall accuracy is high and accuracy measures may be subject to ceiling effects. Finally, the present study focused on MD as the primary trajectory index. More fine-grained temporal measures, such as velocity profiles or the latency of maximum deviation, may provide additional information about when reward-driven competition emerges and how it is corrected during movement. These analyses were beyond the scope of the present validation study but represent useful directions for future work.

Taken together, the findings indicate that mouse tracking can serve as a complementary behavioral tool for studying reward-driven attentional bias and its individual-difference correlates. Rather than replacing RT or eye-tracking measures, this approach adds a process-level index of how reward history shapes the unfolding competition between attention and action.

## Figures and Tables

**Table 1:** Questionnaires used in the present study.

Sample	Measure	Version	Items	Response Scale	Scoring	Cronbach’s α
S1 ^1^	Internet Addiction Test (IAT)	(Young, 1998)	20	5-point Likert (1–5; 1 = not applicable, 5 = always)	Total score = sum of all items, scores range 20–100. Higher scores indicate greater severity.	0.90
S1	Social Media Disorder Scale (SMD)	(Van Den Eijnden et al., 2016)	9	Dichotomous (Yes = 1/No = 0)	Endorsing ≥ 5 criteria indicates SMD.	0.77
S1 & S2 ^2^	DSM-5 Internet Gaming Disorder	(American Psychiatric Association, 2013)	9	Dichotomous (Yes = 1/No = 0)	Endorsing ≥ 5 criteria indicates IGD.	0.89
S2	IGD-20 test	(Yu et al., 2019) (Chinese version)	20	5-point Likert (1–5; 1 = strongly disagree, 5 = strongly agree)	Total score = sum of all items, items 2 and 9 are reversely scored. Scores range 20–100, with higher scores indicating greater severity.	0.88
S2	Short Video Indulgence Scale (SVIS)	(H. Chen et al., 2025)	17	5-point Likert (1–5; 1 = never, 5 = always)	Total score = sum of all items, Scores range 17–85, with higher scores indicating greater severity.	0.92
S2	DSM-5 Short video version	(American Psychiatric Association, 2013)	9	Dichotomous (Yes = 1/No = 0)	In this version, we substitute the “Internet gaming” with “short video”.	0.89
S1 & S2	Sensitivity to Punishment and Sensitivity to Reward Questionnaire (SPSRQ)	(Wang, 2012) (Chinese version)	34	Dichotomous (Yes = 1/No = 0)	Calculate total scores for the two subscales: Sensitivity to Reward (SR, 16 items) and Sensitivity to Punishment (SP, 18 items). SR scores range 0–16, SP 0–18; higher scores indicate greater sensitivity (reward for SR, punishment for SP).	SR: 0.68
S1 & S2	Self-Control Scale (SCS)	(Tan & Guo, 2008) (Chinese version)	19	5-point Likert (1–5; 1 = not at all, 5 = very much)	Total score = sum of all items; items 2, 3, 4, 6, 7, 8, 9, 10, 12, 13, 15, 16, 17, 18, 19 are reversely scored. Scores range 19–95, with higher scores indicating greater self-control.	0.83
S1 & S2	Barratt Impulsiveness Scale (BIS-11)	(Li et al., 2011) (Chinese version)	30	5-point Likert (1–5; 1 = never, 5 = always)	Calculate total scores for the three subscales (10 items each): Attentional, Motor, and Non-planning. Attentional and Non-planning are reverse-scored. Each subscale scores range 10–50. Scores were linearly transformed to a 0–100 scale for each subscale, with higher scores indicating greater impulsivity.	0.74

^1^ S1 = Initial sample ^2^ S2 = Replication sample.

**Table 2:** Correlation Coefficients and Corresponding *p*-values Between Each of ΔRT and ΔMD and Questionnaire Scores in the Initial Sample.

Questionnaires	ΔRT (ms)			ΔMD (Deg)		
	*r* ^1^	*p*	95%CI	*r* ^1^	*p*	95%CI
IAT	0.081	0.595	[−0.217, 0.366]	0.376	0.011	[0.093, 0.603]
DSM-5	0.190	0.212	[−0.110, 0.458]	0.354	0.017	[0.067, 0.587]
SMD	−0.077	0.616	[−0.362, 0.222]	0.025	0.868	[−0.270, 0.317]
PCA (addiction tendency)	0.080	0.601	[−0.219, 0.365]	0.317	0.034	[0.026, 0.559]
SR	0.253	0.094	[−0.044, 0.508]	0.417	0.004	[0.140, 0.633]
SCS	−0.092	0.548	[−0.375, 0.207]	−0.331	0.026	[−0.569, −0.041]
BIS-11	0.076	0.618	[−0.222, 0.362]	0.297	0.048	[0.003, 0.543]

^1^ *r* = Pearson correlation coefficient.

**Table 3:** Correlation Coefficients and Corresponding *p*-values Between Each of ΔRT and ΔMD and Questionnaire Scores in the Replication Sample.

Questionnaires	ΔRT (ms)			ΔMD (Deg)		
	*r* ^1^	*p*	95%CI	*r* ^1^	*p*	95%CI
SVIS	0.181	0.171	[−0.079, 0.417]	0.262	0.045	[0.006, 0.485]
DSM-5 video	0.112	0.400	[0.149, 0.357]	0.173	0.190	[−0.087, 0.411]
IGD-20	0.158	0.232	[−0.102, 0.398]	0.299	0.022	[0.046, 0.515]
DSM-5 game	0.204	0.121	[−0.055, 0.437]	0.339	0.009	[0.091, 0.548]
PCA (addiction tendency)	0.220	0.095	[−0.039, 0.450]	0.361	0.005	[0.116, 0.565]
SR	0.142	0.285	[−0.119, 0.384]	0.422	<0.001	[0.186, 0.612]
SCS	−0.217	0.099	[0.448, 0.041]	−0.426	<0.001	[−0.615, −0.191]
BIS-11	0.239	0.068	[−0.018, 0.467]	0.301	0.021	[0.048, 0.517]

^1^ *r* = Pearson correlation coefficient.

## Data Availability

The data presented in this study are available on request from the corresponding author due to privacy and ethical restrictions.

## Abbreviations

The following abbreviations are used in this manuscript:

VDAC	Value-Driven Attentional Capture
MD	Maximum Deviation
PCA	Principal Component Analysis
IAT	Internet Addiction Test
SMD	Social Media Disorder Scale
BIS-11	Barratt Impulsiveness Scale
SCS	Self-Control Scale
SR	Sensitivity to Reward
SVIS	Short Video Indulgence Scale
IGD	Internet Gaming Disorder

## References

[B1-behavsci-16-01273] Albertella L., Copeland J., Pearson D., Watson P., Wiers R. W., Le Pelley M. E. (2017). Selective attention moderates the relationship between attentional capture by signals of nondrug reward and illicit drug use. Drug and Alcohol Dependence.

[B2-behavsci-16-01273] Albertella L., Le Pelley M. E., Chamberlain S. R., Westbrook F., Fontenelle L. F., Segrave R., Lee R., Pearson D., Yücel M. (2019). Reward-related attentional capture is associated with severity of addictive and obsessive-compulsive behaviors. Psychology of Addictive Behaviors.

[B3-behavsci-16-01273] Albertella L., Le Pelley M. E., Chamberlain S. R., Westbrook F., Lee R. S. C., Fontenelle L. F., Grant J. E., Segrave R. A., McTavish E., Yücel M. (2020). Reward-related attentional capture and cognitive inflexibility interact to determine greater severity of compulsivity-related problems. Journal of Behavior Therapy and Experimental Psychiatry.

[B4-behavsci-16-01273] Albertella L., Vd Hooven J., Bovens R., Wiers R. W. (2021). Reward-related attentional capture predicts non-abstinence during a one-month abstinence challenge. Addictive Behaviors.

[B5-behavsci-16-01273] American Psychiatric Association (2013). Diagnostic and statistical manual of mental disorders.

[B6-behavsci-16-01273] Anderson B. A. (2013). A value-driven mechanism of attentional selection. Journal of Vision.

[B7-behavsci-16-01273] Anderson B. A. (2016). What is abnormal about addiction-related attentional biases?. Drug and Alcohol Dependence.

[B8-behavsci-16-01273] Anderson B. A. (2021). Relating value-driven attention to psychopathology. Current Opinion in Psychology.

[B9-behavsci-16-01273] Anderson B. A., Faulkner M. L., Rilee J. J., Yantis S., Marvel C. L. (2013). Attentional bias for nondrug reward is magnified in addiction. Experimental and Clinical Psychopharmacology.

[B10-behavsci-16-01273] Anderson B. A., Kim H. (2019). Test–retest reliability of value-driven attentional capture. Behavior Research Methods.

[B11-behavsci-16-01273] Anderson B. A., Kuwabara H., Wong D. F., Gean E. G., Rahmim A., Brasic J. R., George N., Frolov B., Courtney S. M., Yantis S. (2016). The role of dopamine in value-based attentional orienting. Current Biology.

[B12-behavsci-16-01273] Anderson B. A., Laurent P. A., Yantis S. (2011). Value-driven attentional capture. Proceedings of the National Academy of Sciences of the United States of America.

[B13-behavsci-16-01273] Anderson B. A., Leal S. L., Hall M. G., Yassa M. A., Yantis S. (2014). The attribution of value-based attentional priority in individuals with depressive symptoms. Cognitive, Affective & Behavioral Neuroscience.

[B14-behavsci-16-01273] Basel D., Lazarov A. (2023). Reward functioning from an attentional perspective and obsessive-compulsive symptoms-an eye-tracking study. CNS Spectrums.

[B15-behavsci-16-01273] Berridge K. C., Robinson T. E. (1998). What is the role of dopamine in reward: Hedonic impact, reward learning, or incentive salience?. Brain Research Reviews.

[B16-behavsci-16-01273] Chen H., Wang D., Zhang L., Zhou H., Liao Y., Hu Y. (2025). Development and validation of the short video indulgence scale. Chinese Journal of Clinical Psychology.

[B17-behavsci-16-01273] Chen Y., Chen S., Zhang X., Zhang S., Jia K., Anderson B. A., Gong M. (2023). Reward history modulates attention based on feature relationship. Journal of Experimental Psychology: General.

[B18-behavsci-16-01273] Dalmaso M. (2022). Exploring the social environment with the eyes: A review of the impact of facial stimuli on saccadic trajectories. International Journal of Environmental Research and Public Health.

[B19-behavsci-16-01273] Dayan P. (2009). Dopamine, reinforcement learning, and addiction. Pharmacopsychiatry.

[B20-behavsci-16-01273] Dong G., Potenza M. N. (2014). A cognitive-behavioral model of Internet gaming disorder: Theoretical underpinnings and clinical implications. Journal of Psychiatric Research.

[B21-behavsci-16-01273] Draheim C., Mashburn C. A., Martin J. D., Engle R. W. (2019). Reaction time in differential and developmental research: A review and commentary on the problems and alternatives. Psychological Bulletin.

[B22-behavsci-16-01273] Egner S., Reimann S., Hoeger R., Zangemeister W. H. (2018). Attention and information acquisition: Comparison of mouse-click with eye-movement attention tracking. Journal of Eye Movement Research.

[B23-behavsci-16-01273] Faul F., Erdfelder E., Lang A. G., Buchner A. (2007). G*Power 3: A flexible statistical power analysis program for the social, behavioral, and biomedical sciences. Behavior Research Methods.

[B24-behavsci-16-01273] Flagel S. B., Robinson T. E. (2017). Neurobiological basis of individual variation in stimulus-reward learning. Current Opinion in Behavioral Sciences.

[B25-behavsci-16-01273] Freeman J. B., Ambady N. (2010). MouseTracker: Software for studying real-time mental processing using a computer mouse-tracking method. Behavior Research Methods.

[B26-behavsci-16-01273] Freichel R., Mrkonja L., de Jong P. J., Cousijn J., Franken I., Ruiter T. A., Le Pelley M., Albertella L., Watson P., Veer I. M., Wiers R. W. (2023). Value-modulated attentional capture in reward and punishment contexts, attentional control, and their relationship with psychopathology. Journal of Experimental Psychopathology.

[B27-behavsci-16-01273] Garre-Frutos F., Vadillo M. A., González F., Lupiáñez J. (2024). On the reliability of value-modulated attentional capture: An online replication and multiverse analysis. Behavior Research Methods.

[B28-behavsci-16-01273] Hehman E., Stolier R. M., Freeman J. B. (2015). Advanced mouse-tracking analytic techniques for enhancing psychological science. Group Processes & Intergroup Relations.

[B29-behavsci-16-01273] Hickey C., Chelazzi L., Theeuwes J. (2010). Reward changes salience in human vision via the anterior cingulate. Journal of Neuroscience.

[B30-behavsci-16-01273] Holroyd C. B., Coles M. G. H. (2002). The neural basis of human error processing: Reinforcement learning, dopamine, and the error-related negativity. Psychological Review.

[B31-behavsci-16-01273] Hyman S. E., Malenka R. C., Nestler E. J. (2006). Neural mechanisms of addiction: The role of reward-related learning and memory. Annual Review of Neuroscience.

[B32-behavsci-16-01273] Keiflin R., Janak P. H. (2015). Dopamine prediction errors in reward learning and addiction: From theory to neural circuitry. Neuron.

[B33-behavsci-16-01273] Kieslich P. J., Henninger F. (2017). Mousetrap: An integrated, open-source mouse-tracking package. Behavior Research Methods.

[B34-behavsci-16-01273] Kim S. H., Yoon H., Kim H., Hamann S. (2015). Individual differences in sensitivity to reward and punishment and neural activity during reward and avoidance learning. Social Cognitive and Affective Neuroscience.

[B35-behavsci-16-01273] Le Pelley M. E. (2016). Attention and associative learning in humans: An integrative review. Trends in Cognitive Sciences.

[B36-behavsci-16-01273] Le Pelley M. E., Pearson D., Griffiths O., Beesley T. (2015). When goals conflict with values: Counterproductive attentional and oculomotor capture by reward-related stimuli. Journal of Experimental Psychology: General.

[B37-behavsci-16-01273] Li X. Y., Phillips M. R., Xu D., Zhang Y. L., Yang S. J., Tong Y. S., Wang Z.-Q., Niu Y. J. (2011). Reliability and validity of an adapted Chinese version of Barratt Impulsiveness Scale. Chinese Mental Health Journal.

[B38-behavsci-16-01273] Meyer T., Kim A. D., Spivey M., Yoshimi J. (2023). Mouse tracking performance: A new approach to analyzing continuous mouse tracking data. Behavior Research Methods.

[B39-behavsci-16-01273] Mollick J. A., Chang L. J., Krishnan A., Hazy T. E., Krueger K. A., Frank G. K. W., Wager T. D., O’Reilly R. C. (2021). The neural correlates of cued reward omission. Frontiers in Human Neuroscience.

[B40-behavsci-16-01273] O’Doherty J. P., Cockburn J., Pauli W. M. (2017). Learning, reward, and decision making. Annual Review of Psychology.

[B41-behavsci-16-01273] Patton J. H., Stanford M. S., Barratt E. S. (1995). Factor structure of the Barratt impulsiveness scale. Journal of Clinical Psychology.

[B42-behavsci-16-01273] Pearson D., Watson P., Albertella L., Le Pelley M. E. (2022). Attentional economics links value-modulated attentional capture and decision-making. Nature Reviews Psychology.

[B43-behavsci-16-01273] Pontes H. M., Király O., Demetrovics Z., Griffiths M. D. (2014). The conceptualisation and measurement of DSM-5 Internet Gaming Disorder: The development of the IGD-20 Test. PLoS ONE.

[B44-behavsci-16-01273] Ran X., Wang M., Huang J., Kuang N., Tian P., Wu J., Feng F., Luo Y., Huang N. (2026). Mechanistic research and therapeutic prospects of alternative splicing in neurodegenerative diseases. Ageing Research Reviews.

[B45-behavsci-16-01273] Robinson T. E., Berridge K. C. (1993). The neural basis of drug craving: An incentive-sensitization theory of addiction. Brain Research Reviews.

[B46-behavsci-16-01273] Rusz D., Le Pelley M. E., Kompier M. A. J., Mait L., Bijleveld E. (2020). Reward-driven distraction: A meta-analysis. Psychological Bulletin.

[B47-behavsci-16-01273] Sali A. W., Anderson B. A., Yantis S., Mostofsky S. H., Rosch K. S. (2018). Reduced value-driven attentional capture among children with ADHD compared to typically developing controls. Journal of Abnormal Child Psychology.

[B48-behavsci-16-01273] Schultz W. (1998). Predictive reward signal of dopamine neurons. Journal of Neurophysiology.

[B49-behavsci-16-01273] Schultz W. (2016). Dopamine reward prediction-error signalling: A two-component response. Nature Reviews Neuroscience.

[B50-behavsci-16-01273] Shih C. Y., Li S. H., Ho M. C. (2025). Single scores in the RT-based value-driven attentional capture paradigm have high reliability. Scientific Reports.

[B51-behavsci-16-01273] Smith K. A., Morrison S., Henderson A. M., Erb C. D. (2022). Moving beyond response times with accessible measures of manual dynamics. Scientific Reports.

[B52-behavsci-16-01273] Song J.-H., Nakayama K. (2009). Hidden cognitive states revealed in choice reaching tasks. Trends in Cognitive Sciences.

[B53-behavsci-16-01273] Spivey M. J., Dale R. (2006). Continuous dynamics in real-time cognition. Current Directions in Psychological Science.

[B54-behavsci-16-01273] Spivey M. J., Grosjean M., Knoblich G. (2005). Continuous attraction toward phonological competitors. Proceedings of the National Academy of Sciences of the United States of America.

[B55-behavsci-16-01273] Stanković M. (2025). Systematic review of the value-driven attentional capture paradigm in visual attention studies: Evidence from 52 experiments. Acta Psychologica.

[B56-behavsci-16-01273] Starcke K., Antons S., Trotzke P., Brand M. (2018). Cue-reactivity in behavioral addictions: A meta-analysis and methodological considerations. Journal of Behavioral Addictions.

[B57-behavsci-16-01273] Tan S. H., Guo Y. Y. (2008). Revision of self-control scale for Chinese college students. Chinese Journal of Clinical Psychology.

[B58-behavsci-16-01273] Tangney J. P., Baumeister R. F., Boone A. L. (2004). High self-control predicts good adjustment, less pathology, better grades, and interpersonal success. Journal of Personality.

[B59-behavsci-16-01273] Theeuwes J., Belopolsky A. V. (2012). Reward grabs the eye: Oculomotor capture by rewarding stimuli. Vision Research.

[B60-behavsci-16-01273] Torrubia R., Avila C., Moltó J., Caseras X. (2001). The Sensitivity to Punishment and Sensitivity to Reward Questionnaire (SPSRQ) as a measure of Gray’s anxiety and impulsivity dimensions. Personality and Individual Differences.

[B61-behavsci-16-01273] Van Den Eijnden R. J., Lemmens J. S., Valkenburg P. M. (2016). The social media disorder scale. Computers in Human Behavior.

[B62-behavsci-16-01273] Wang E. J. (2012). Reliability and validity of Chinese version of SPSRQ in university students. Chinese Journal of School Health.

[B63-behavsci-16-01273] Whitton A. E., Treadway M. T., Pizzagalli D. A. (2015). Reward processing dysfunction in major depression, bipolar disorder and schizophrenia. Current Opinion in Psychiatry.

[B64-behavsci-16-01273] Young K. S. (1998). Internet addiction: The emergence of a new clinical disorder. Cyberpsychology & Behavior.

[B65-behavsci-16-01273] Yu S. M., Pesigan I. J. A., Zhang M. X., Wu A. M. S. (2019). Psychometric validation of the Internet Gaming Disorder-20 Test among Chinese middle school and university students. Journal of Behavioral Addictions.

